# Decline in mean platelet volume in patients with patent foramen ovale undergoing percutaneous closure

**DOI:** 10.5830/CVJA-2014-027

**Published:** 2014

**Authors:** Bariş Düzel, Nihan Kahya Eren, Uğur Kocabaş, Mustafa Gönençer, Cem Nazli, Oktay Ergene, Rida Berilgen

**Affiliations:** Mersin State Hospital, Mersin, Turkey; Izmir Katip Çelebi University, Cardiology Clinic, Atatürk Research and Education Hospital, Turkey; Izmir Katip Çelebi University, Cardiology Clinic, Atatürk Research and Education Hospital, Turkey; Izmir Katip Çelebi University, Cardiology Clinic, Atatürk Research and Education Hospital, Turkey; Izmir Katip Çelebi University, Cardiology Clinic, Atatürk Research and Education Hospital, Turkey; Izmir Katip Çelebi University, Cardiology Clinic, Atatürk Research and Education Hospital, Turkey; Mardin Kiziltepe State Hospital, Mardin, Turkey

**Keywords:** patent foramen ovale, ischaemic stroke, platelets, transcatheter closure

## Abstract

**Introduction:**

The presence of patent foramen ovale (PFO) is considered a possible cause for cryptogenic stroke. The mechanism underlying the ischaemic neurological events in the presence of PFO has not been firmly established. The purpose of this study was to compare: (1) the mean platelet volume levels in PFO patients with and without a cryptogenic stroke, and (2) pre- and post-procedural mean platelet volumes (MPV) in patients undergoing percutaneous PFO closure.

**Methods:**

Sixteen PFO patients undergoing percutaneous closure to prevent recurrent ischaemic events and 15 asymptomatic patients with PFO were enrolled in the study. Mean platelet volume was compared between patients with and without a history of stroke. We also compared pre- and postprocedural MPV levels in patients undergoing percutaneous PFO closure.

**Results:**

Mean platelet volume, which is a marker for platelet activity, was similar in PFO patients with and without stroke (9.34 ± 1.64 vs 9.1 ± 1.34 fl; *p* = 0.526). Interestingly, MPV decreased significantly after percutaneous closure compared to pre-procedural levels (9.34 ± 1.64 vs 8.3 ± 1.12 fl; *p* = 0.001).

**Conclusion:**

Our findings suggest interatrial communication through a PFO may be related to increased MPV and increased platelet activity.

## Abstract

Patent foramen ovale (PFO) is a haemodynamically insignificant communication that is present in 24% of the general population.^1^ In 1988 Lechat *et al.* performed transthoracic echocardiography (TTE) with contrast injection and showed that patients with stroke of unknown cause had PFOs more frequently than the controls.^2^ Since then, many studies have confirmed this association. In 2000, a meta-analysis summarised the evidence that PFO was more likely to be found in stroke patients than in stroke-free individuals.^3^

In about 50 to 60% of patients younger than 55 years, the cause of acute ischaemic stroke remains undefined.^4^ In this group, interatrial septal abnormalities are found in 55 to 60% of cases, which is higher than in the normal population.

In another meta-analysis, Mattle et al. reported a higher prevelance of PFO in patients with cryptogenic stroke than in patients with a stroke of known causes.^1^ The postulated possible mechanisms underlying the stroke in the presence of PFO are: paradoxycal embolism, thrombus formation within the conduit of the PFO, or susceptibility of patients with PFO to atrial arrhythmias with possible intra-atrial thrombus formation.^4^-^8^

Although paradoxycal embolism, which is associated with deep-vein thrombosis (DVT), is the favoured hypothesis, DVT in patients with PFO is usually undetectable.^9^ Therefore, increased platelet activity as well as disorders in the coagulation cascade may contribute to the association between PFO and stroke.

Mean platelet volume (MPV) is a measure of platelet size and is a potential marker of platelet reactivity. It has been shown that larger platelets are metabolically and enzymatically more active and have greater prothombotic potential.^10^,^11^

The aim of this study was (1) to compare MPVs of PFO patients with and without a history of cryptogenic stroke, and (2) to determine the effect of percutaneous PFO closure on MPV.

## Methods

Between January 2008 and June 2012, 16 consecutive patients who had suffered cryptogenic stroke and underwent percutaneous PFO closure to prevent recurrent cerebral ischaemic events, and 15 consecutive patients with a diagnosis of PFO but without a history of stroke were recruited into the study. The diagnosis of PFO was established if any microbubble was seen in the left-sided cardiac chambers within three cardiac cycles from the maximum right atrial opacification after contrast injection during transoesophageal echocardiography (TEE).

Demographic data and history of conventional risk factors such as smoking habits, hypertension, diabetes mellitus, hyperlipidaemia, history of vascular disease (stroke, coronary artery disease, peripheral artery disease) and medications were recorded for every patient.

Non-fasting blood samples were taken from each patient to assess mean platelet volume and standard blood tests. In patients who underwent percutaneous PFO closure, mean platelet volume was measured before percutaneous PFO closure and at the six-month follow up after the intervention. In patients with PFO without a history of stroke, MPV was measured once at the time of enrollment.

Percutaneous PFO closure was performed under fluoroscopic and TEE guidance and under general anaesthesia. Procedural success was defined as successful implantation of the occluder at the closure site with no procedural complications. Dual antiplatelet therapy including 75 mg clopidogrel for three months and 100 mg aspirin lifelong was recommended after the closure procedure. Patients in the intervention group were followed up at one, three, six and 12 months after the procedure and closure rates and residual shunt were evaluated at the six-month follow up by contrast transthroracic echocardiography.

## Statistical analysis

Continuous variables are presented as mean ± standard deviation and categorical variables are presented as percentages. Group differences for continuous variables were examined by the Mann–Whitney *U*-test. For categorical variables, comparisons between groups were made with the χ^2^ or Fisher exact test, as appropriate. Pre- and post-procedural MPV levels in the intervention group were compared with the Wilcoxon signed-rank test.

## Results

A total of 31 patients were enrolled in the study. Baseline demographic and clinical characteristics of all patients are presented in Table 1. The patients in the intervention group were significantly older than those without a history of stroke (48.25 ± 12.1 vs 37.4 ± 15.32 years; p = 0.028). The other baseline characteristics were similar between the two groups. The mean age of the patients in the intervention group at the time of their first ischaemic attack was 45.38 ± 12.01 years.

**Table 1:** Demographic and clinical characteristics of the patients

	*PFO patients with stroke*	*PFO patients without stroke*	p*-value*
Number	16	15	
Male (%)	44	13	0.113
Age (years)	48.2 ± 12.1	37.4 ± 15.3	0.028
Current smoker (%)	44	47	0.870
Hypertension (%)	19	7	0.600
Diabetes mellitus (%)	25	0	0.101
Hyperlipidaemia (%)	6	0	1.00
Coronary artery disease (%)	0	0	1.00
Antiplatelet use at baseline (%)	44	0	
Oral anticoagulan therapy at baseline (%)	56	0	
MPV at baseline (fl)	9.34 ± 1.64	9.10 ± 1.34	0.526
Platelet count at baseline	288562 ± 80180	267400 ± 56368	0.527

Procedural success rate was 100% in the intervention group. Cardio Fix, BioSTAR, Amplatzer and Cardioseal/Starflex devices were implanted in eight, six, one and one patients, respectively. Residual shunt was detected in three (19%) patients at the six-month follow up. None of the patients suffered a recurrent ischaemic attack after the intervention during a mean follow-up period of 26.5 ± 1.3 months.

Forty-four per cent of the patients undergoing PFO closure were receiving aspirin (100 mg/day), and the remainder (56%) were taking oral anticoagulant therapy before the procedure. After PFO closure, all patients were prescribed dual antiplatelet therapy with asetylsalicilic asid (100 mg/day) and clopidogrel (75 mg/day).

**Fig. 1.**
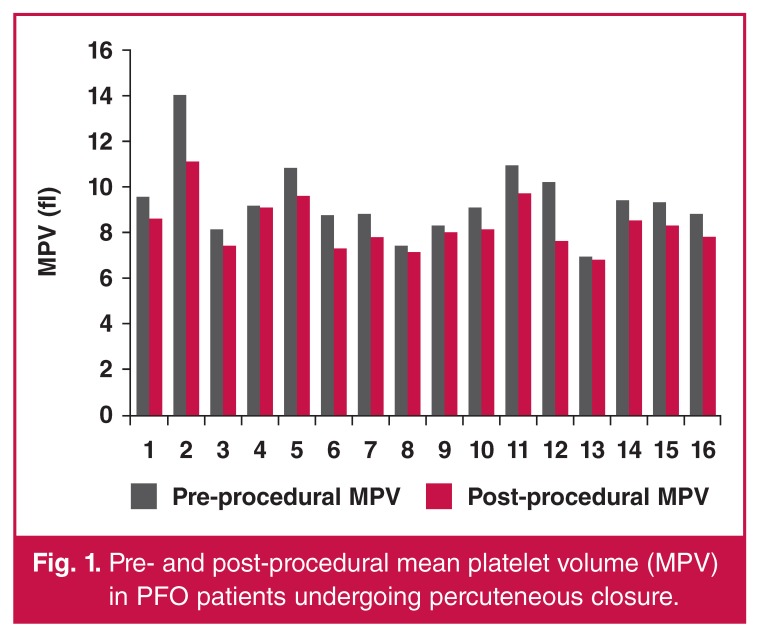
Pre- and post-procedural mean platelet volume (MPV) in PFO patients undergoing percuteneous closure.

The patients with PFO and cryptogenic stroke had similar mean platelet volumes to the patients with PFO but without stroke (9.34 ± 1.64 fl vs. 9.1 ± 1.34 fl; *p* = 0.526). On the other hand, we observed a significant decline in MPV after transcatheter PFO closure in patients with stroke compared with their pre-procedural levels (9.34 ± 1.64 fl vs 8.3 ± 1.12 fl; *p* = 0.001). Pre- and post-procedural changes in MPV in patients undergoing percutaneous closure is represented in Fig. 1.

There was no difference in platelet count in patients with and without stroke. There were also no significant differences in pre-and post-closure platelet count levels in the intervention group (Table 1).

## Discussion

We presumed that platelet activity may be increased in PFO patients with stroke compared to those without stroke, but we did not find a difference between these two groups. Interestingly, we observed that MPV levels decreased after percutaneous PFO closure in patients with previous stroke compared with their pre-procedural levels.

Mean platelet volume is a measure of platelet size, and is a determinant of platelet activity. Larger platelets are metabolically and enzymatically more active than smaller platelets, containing more prothrombotic material such as thromboxane A2 and serotonin, and expressing greater numbers of adhesion molecules such as Glp IIb/IIIa receptor and P-selectin.^11^-^14^ They also show greater aggregation in response to ADP.10 Previous studies have shown that MPV was increased in myocardial infarction and ischaemic stroke, both of which are atherothrombotic events where platelets play a pivotal role.

The association of PFO with cryptogenic stroke has been established previously,^1^,^4^ but the pathogenic link between PFO and stroke is still unclear in most cases. One of the postulated mechanisms underlying the stroke in the presence of PFO is paradoxycal embolism with an occult DVT. Although some studies have shown increased frequency of prothrombotic mutations, which may be a predisposing factor for the development of DVT in patients with PFO, other studies found no difference between PFO patients and controls.^15^-^18^

Accordingly, the majority of strokes associated with PFO cannot be explained by paradoxycal embolism. We hypothesised that increased platelet activity may be a factor in the pathogenesis of cryptogenic stroke in patients with PFO, and found that PFO patients with and without stroke had similar MPV, which was decreased after percutaneous closure.

Increased platelet activity in patients with right-to-left shunt may be a precipitating factor in thrombus formation in the conduit of PFO, which is one of the proposed pathogenic links between PFO and stroke. The absence of recurrent neurological events during the mean follow-up period of 26.5 ± 1.3 months in the intervention group may also suggest that decreased MPV and platelet activity may have a possible protective role for recurrent ischaemic events after the closure procedure. However, why patients with right-to-left shunt have increased platelet activity is a dilemma.

Forty-four per cent of patients in the intervention group were receiving aspirin before the procedure, after which dual antiplatelet therapy was prescribed to all patients for three months. Finally, aspirin monotherapy was recommended lifelong. Although there are limited data regarding the effect of antiplatelet agents on MPV, aspirin does not seem to significantly affect platelet volume.^19^,^20^ Likewise, large platelets exhibit increased reactivity even after dual antiplatelet therapy in patients with stable coronary artery disease.^21^ These data support the hypothesis that post-procedural decline in MPV cannot be solely attributed to a higher rate of usage of antiplatelet therapy after the intervention.

This study is limited by its small sample size. The lack of significant difference in MPV between patients with and without stroke may be a type 2 error due to insufficient sample size. Mean platelet volume may be greater in patients with cryptogenic stroke and PFO compared with asymptomatic patients with PFO. This should be clarified in further randomised studies with larger patient populations.

Additionally, the observed post-procedural decline in MPV may be related to factors such as different rates of pre- and post-procedural antiplatelet and anticoagulant therapy usage as well as the PFO closure device itself. Therefore, performing other laboratory tests in addition to platelet count and MPV to estimate platelet activity would have strenghtened our results.

On the other hand, this is the first study to our knowledge that demonstrates a decrease in platelet activity in patients with PFO undergoing percutaneous closure. Our findings warrant further studies to investigate the platelet activity in PFO patients as a risk factor for cryptogenic stroke and its possible association with percutaneous closure.

## Conclusion

Our findings suggest interatrial communication through a PFO may be related to increased MPV and increased platelet activity.
